# Unicentric Castleman disease of plasma cell type in the adrenal region: a case report and literature review

**DOI:** 10.3389/fonc.2026.1893723

**Published:** 2026-08-18

**Authors:** Wei Gao, Mingkun Hu, Xiang Dai, Guobiao Liu, Dongxia Liu, Shengjun Liang, Nan Jin, Jing Lu

**Affiliations:** 1 Department of Urology, Liwan Central Hospital of Guangzhou, Guangzhou, China; 2 First Affiliated Hospital of Guangzhou Medical University, Guangzhou, China

**Keywords:** adrenal neoplasm, Castleman’s disease, differential diagnosis, plasma cell type, surgical resection

## Abstract

**Background:**

Castleman disease (CD) comprises a heterogeneous group of lymphoproliferative disorders. Unicentric CD (UCD) involving the adrenal region is uncommon and poses diagnostic challenges because it can resemble adrenal neoplasms.

**Case presentation:**

A 37-year-old female presented with a 10-year history of intermittent hyperhidrosis and facial flushing, and a one-month history of right chest and abdominal pain. Imaging revealed a 53 mm × 42 mm heterogeneous mass in the left adrenal region with calcification and marked enhancement. Endocrine evaluation showed no hormonal hypersecretion. The patient underwent laparoscopic tumor resection. Histopathology and immunohistochemistry confirmed plasma cell type UCD, with HHV-8 negative on both serology and immunohistochemistry. Postoperative recovery was uneventful. Postoperative PET/CT and PET/MRI showed no evidence of residual or recurrent disease. At the two-year follow-up, the patient remained asymptomatic with no evidence of recurrence.

**Conclusion:**

Adrenal-region UCD is an uncommon entity that can mimic nonfunctional adrenal tumors. Complete surgical excision is curative. This case highlights the importance of including UCD in the differential diagnosis of well-defined adrenal masses without endocrine abnormalities.

## 1 Introduction

Castleman disease (CD) comprises a heterogeneous group of disorders involving a broad lymph node histopathological spectrum and is clinically classified as unicentric Castleman disease (UCD), multicentric Castleman disease (MCD), and an oligocentric form that has also been recognized clinically (1, 2). UCD, which involves a single enlarged lymph node or multiple enlarged lymph nodes within a single lymph node station, was first described by Castleman and Towne (3) in a patient with a mediastinal mass in 1954 and soon thereafter by Castleman et al. in a series of 12 cases in 1956 (4). Histopathologic variants of CD include hyaline vascular type, Plasma cell type, and mixed form (5). The etiology of CD is still unclear and is associated with dysregulated interleukin-6 (IL-6) signaling and the JAK-STAT/mTOR pathway (6, 7). CD can affect lymph nodes in any anatomical region, most frequently involving the mediastinum, cervical, retroperitoneal, and axillary regions. The adrenal region accounts for approximately 4%-12% of UCD cases (8). UCD is usually detected incidentally on radiological imaging or presents as a symptomatic lymph node mass. However, preoperative diagnosis of UCD is often difficult, as pathological confirmation is required (9).

The first manifestation is usually the discovery of a slow-growing tumor or systemic symptoms such as fever and weight loss, and the duration of systemic symptoms or lymph node enlargement varies from several weeks to several months. UCD in the adrenal region is rare and is often misdiagnosed as pheochromocytoma or adrenocortical carcinoma preoperatively because of its large size and the nonspecific symptoms and laboratory findings (10). Most UCD cases (74%–91%) show hyaline vascular type histology, while a smaller proportion (9%–26%) show Plasma cell type histology (11). A single-center study from Asia on retroperitoneal unicentric Castleman disease showed that only 4% of cases had Plasma cell type histopathology (9). This article presents a case of plasma cell Plasma cell type UCD arising in the adrenal region, and discusses the clinical manifestations, laboratory findings, imaging and pathological features, treatment, and follow-up of this rare disease, aiming to improve clinicians’ awareness of this rare disorder and avoid misdiagnosis and inappropriate management. A recent case report by Wang Z et al. (2025) described a hyaline vascular type UCD in the left adrenal region (12); the present case differs in its plasma cell histology, systemic inflammatory profile, and long-term follow-up data.

## 2 Case report

### 2.1 Patient presentation and workup

A 37-year-old female presented with a 10-year history of intermittent hyperhidrosis and facial flushing of varying intensity, for which she had not sought medical attention. One month prior to admission, she developed persistent dull pain in the right chest and abdominal regions, without radiation, fever, nausea, vomiting, or weight loss. She presented to our hospital in January 2024. Her medical history was significant for prior hysterectomy, with no history of chronic hypertension, diabetes mellitus, or hepatitis. Abdominal CT revealed a heterogeneous mass (53 mm × 42 mm) in the left adrenal region with well-defined borders, containing linear and punctate calcifications. Additional findings included multiple retroperitoneal nodules and splenomegaly (longest diameter 123.5 mm). Contrast-enhanced CT demonstrated persistent marked enhancement of the lesion during arterial and venous phases, with feeding vessels. The left adrenal gland was compressed and displaced inferiorly, with clear demarcation from the mass. The surrounding fat plane remained clear.

Vital signs were within normal limits: body temperature 36.9 °C, pulse 90 beats/min, blood pressure 125/70 mmHg, respiratory rate 18 breaths/min. The patient was conscious and in good general condition. No enlargement of superficial lymph nodes was detected. Lungs: clear to auscultation bilaterally, without crackles or rhonchi. Heart: regular rate and rhythm, no murmurs appreciated over any valvular area. Abdomen: soft, non-tender, without rebound tenderness. Liver and spleen were not palpable below the costal margins. No masses were palpated in the abdomen or flanks. No costovertebral angle tenderness was elicited bilaterally.

**Figure 1:**
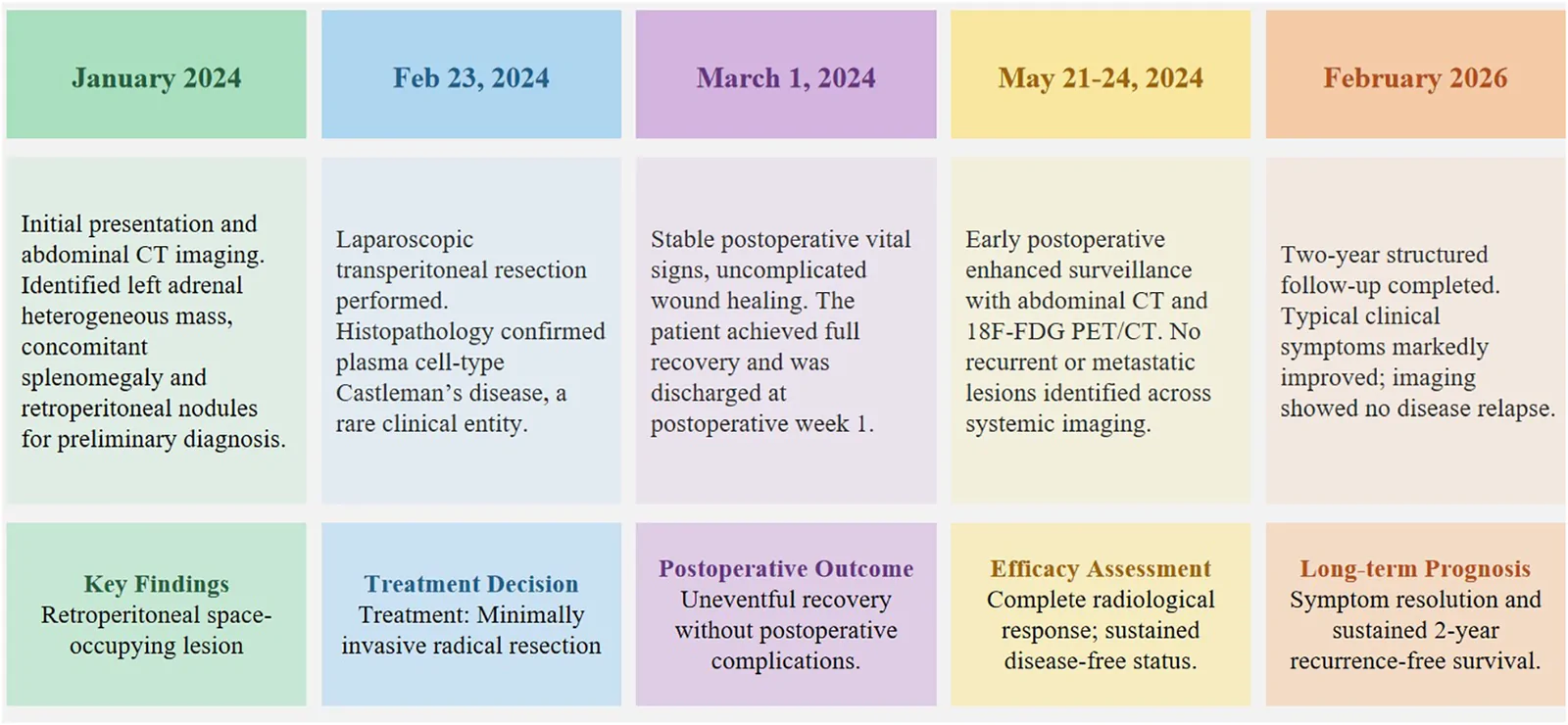
Timeline and diagnostic-therapeutic workflow.

The patient was initially referred to the Department of General Surgery; however, following the detection of an adrenal mass, she was transferred to the Department of Urology. The overall diagnostic and therapeutic pathway of this patient is summarized in **Figure 1**.

### 2.2 Laboratory and endocrine findings

Laboratory and endocrine results are summarized in **Table 1**. Complete blood count parameters, including white blood cell count, hemoglobin, and platelet count, were within normal limits. Liver and renal function tests were unremarkable except for mildly decreased creatinine (38 μmol/L; reference range 40–106 μmol/L). Endocrine evaluation showed no evidence of adrenal hormone hypersecretion, effectively ruling out pheochromocytoma and other functional adrenal tumors. Anti-Müllerian hormone (AMH) was mildly decreased. Preoperative cortisol levels, adrenocorticotropic hormone (ACTH), and renin-angiotensin-aldosterone system (RAAS) parameters (aldosterone, renin, angiotensin II) were all within normal limits, with no evidence of adrenal cortical hyperfunction or insufficiency. Immunological assessment demonstrated elevated erythrocyte sedimentation rate (ESR) and interleukin-6 (IL-6) levels. The Tumor marker carbohydrate antigen 724 (CA724) was mildly elevated. HIV serology was negative. Serum HHV-8 testing was also negative, consistent with the negative HHV-8 immunohistochemistry result. All other laboratory parameters were within reference ranges.

**Table 1:** Summary of laboratory and endocrine findings.

Category	Parameter	Result	Reference range
Hematology	White blood cell count	7.81 × 10^9^/L	3.5-9.5 × 10^9^/L
	Hemoglobin	116 g/L	115–150 g/L
	platelet count	326 × 10^9^/L	125-350 × 10^9^/L
Liver	Alanine Aminotransferase (ALT)	13 U/L	0–35 U/L
	Aspartate Aminotransferase (AST)	17 U/L	5–35 U/L
Renal	Creatinine	38 μmol/L ↓	40-106 μmol/L
Endocrine	Aldosterone (ALD)	171.48 pg/mL	/
	Adrenocorticotropic hormone (ACTH)	18.13 pg/mL	/
	Renin	9.06 pg/mL	/
	Angiotensin	117.62 pg/mL	/
	Aldosterone/renin ratio	18.93 pg/mL	0-30
	Anti-Müllerian hormone (AMH)	0.56 ng/mL ↓	1.43-11.6 ng/mL
Immunology	Rheumatoid factor	12 IU/L	0–40 IU/L
	Erythrocyte sedimentation rate (ESR)	27 mm/H ↑	0–20 mm/H
	Immunoglobulins A (IgA)	82 mg/dL	70–374 mg/dL
	Immunoglobulins G (IgG)	1097 mg/dL	680–1445 mg/dL
	Immunoglobulins M (IgM)	244 mg/dL	40–250 mg/dL
	Interleukin-6 (IL-6)	11.21 pg/mL ↑	0-5.9 pg/mL
	Antinuclear antibody (ANA)	<1:80	<1:80
Tumor markers	Alpha-fetoprotein (AFP)	2.44 ng/mL	0–7 ng/mL
	Carcinoembryonic antigen (CEA)	0.49 ng/mL	0–5 ng/mL
	Carbohydrate antigen 199	2.20 U/mL	0–30 ng/mL
	Carbohydrate antigen 724	13.12 U/mL ↑	0–10 ng/mL
	Carbohydrate antigen 50	3.26 U/mL	0–25 ng/mL
Infection	Human Immunodeficiency Virus (HIV)	Negative	Negative
	Syphilis	Negative	Negative
	Serum HHV-8	Negative	Negative

Postoperative laboratory follow-up (May 2024) showed that IL-6 decreased to 2.63 pg/mL (reference range: 0-5.9 pg/mL) and ESR decreased to 3 mm/h (reference range: 0–20 mm/h), both returning to normal. CA724 was not re-examined postoperatively, which represents a limitation of the present case report. Postoperative splenic longest diameter was 125.3 mm, essentially unchanged from the preoperative measurement.

### 2.3 Imaging studies

**Figure 2:**
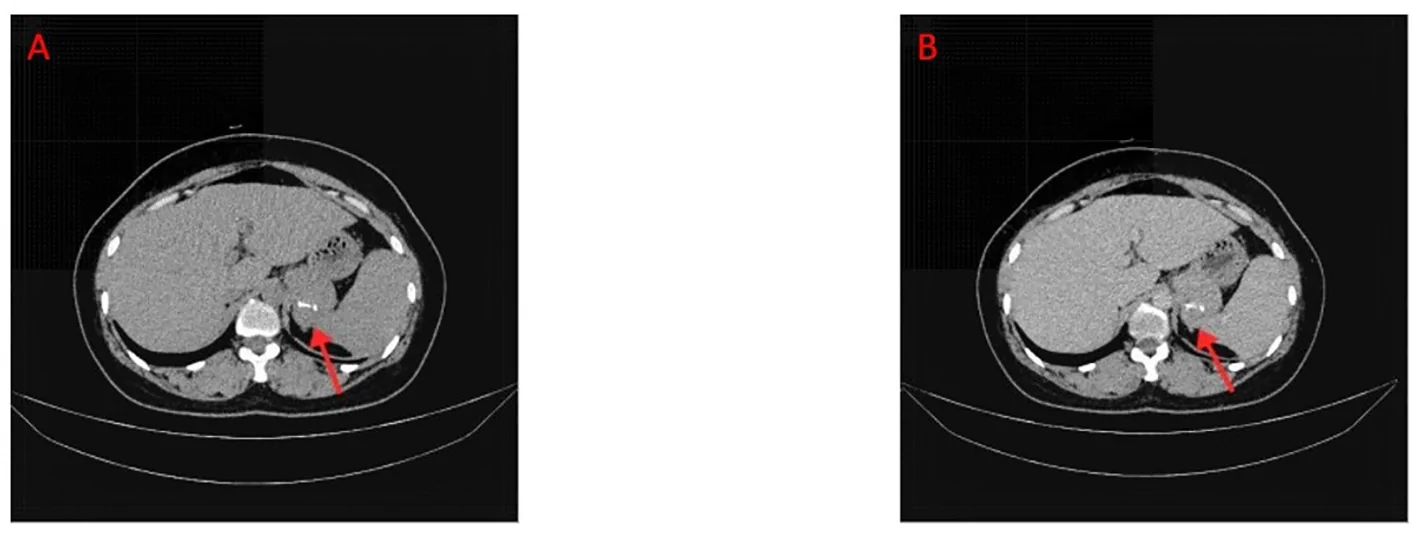
Imaging findings of the left adrenal lesion. **(A)** Plain CT scan: A well-defined heterogeneous mass measuring approximately 53 mm × 42 mm is observed in the left adrenal region, with internal linear and punctate calcifications. **(B)** Contrast-enhanced CT scan: The left adrenal mass presents obvious enhancement. The native left adrenal gland is compressed and displaced downward (arrow).

In the left adrenal region, a well-defined heterogeneous mass measuring approximately 53 mm × 42 mm was observed, with linear and punctate calcifications within it (**Figure 2A**). Contrast-enhanced scan showed persistent marked enhancement of the lesion in both arterial and venous phases, and feeding vessels were noted. The left adrenal gland was compressed and displaced inferiorly, with clear demarcation from the mass (**Figure 2B**). The surrounding fat planes were clear.

Multiple retroperitoneal nodules were seen, the largest measuring about 15 mm × 8 mm, all showing enhancement on contrast scan. Additionally, splenomegaly (longest diameter 123.5 mm) was noted, with no abnormal enhancement in the splenic parenchyma. The uterus was not clearly visualized, consistent with the patient’s history of prior hysterectomy. No other abnormalities were observed.

Based on these imaging features, the preliminary radiological differential diagnosis included a lymphatic or neurogenic tumor; Castleman disease and paraganglioma could not be excluded.

### 2.4 Surgical intervention

**Figure 3:**
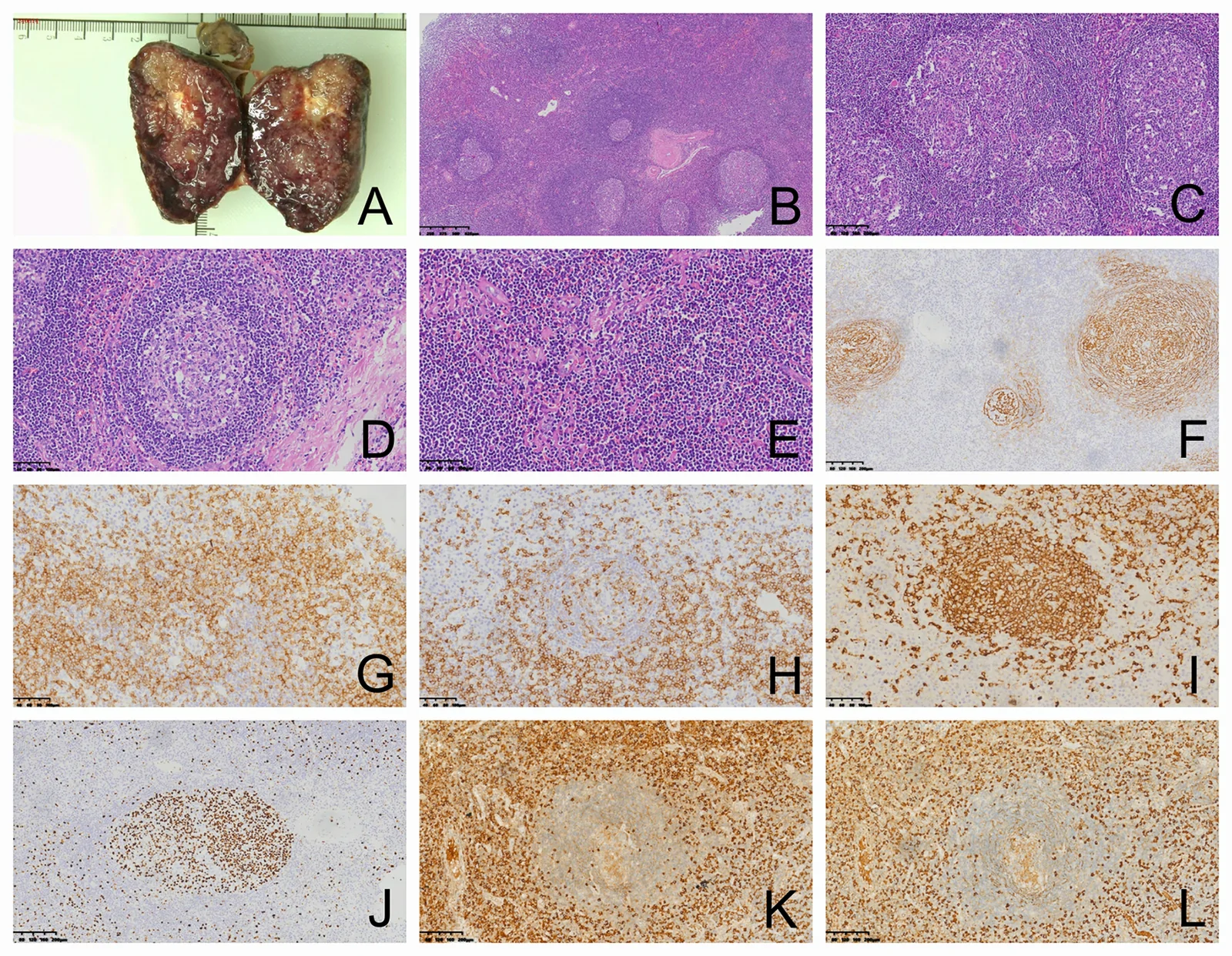
Gross appearance and histopathological as well as immunohistochemical characteristics of the resected lesion. **(A)** Gross specimen: The resected tumor from the left adrenal region presents as a gray-white, firm mass with a smooth surface. **(B–L)** Histopathology and immunohistochemistry of Plasma cell type unicentric Castleman’s disease involving the left adrenal gland. **(B)** H&E staining, ×40: Follicular hyperplasia. **(C)** H&E staining, ×100: Nodular architecture with fibrous stroma and vascular proliferation. **(D)** H&E staining, ×200: Reactive follicles with well-demarcated boundaries. **(E)** H&E staining, ×200: Marked capillary proliferation in the interfollicular areas. **(F)** CD21 immunohistochemistry, ×100: Preserved follicular dendritic cell (FDC) network within germinal centers. **(G)** CD3 immunohistochemistry, ×200: Strong membranous positivity of T cells in the paracortical region. **(H)** CD5 immunohistochemistry, ×200: Lymphocytes arranged in a concentric “onion-skin” pattern around atrophic germinal centers. **(I)** CD20 immunohistochemistry, ×100: Positive staining of B cells in germinal centers, consistent with reactive follicles. **(J)** Ki-67 immunohistochemistry, ×100: High proliferative index in germinal centers with low proliferation in the interfollicular regions. **(K)** κ light chain immunohistochemistry, ×100: Polyclonal plasma cells showing no light chain restriction. **(L)** λ light chain immunohistochemistry, ×100: Polyclonal plasma cells showing no light chain restriction.

After admission, the patient underwent routine preoperative examinations, and no contraindications for surgery were found. On February 23, 2024, laparoscopic transperitoneal retroperitoneal tumor resection was performed. During the operation, the left adrenal gland was exposed. A round, solid lesion measuring approximately 5 cm × 4 cm was identified between the upper medial part of the adrenal gland and the gastric fundus (**Figure 3A**). The lesion adhered to the gastric fundus and Gerota’s fascia. An ultrasonic scalpel was used to dissect the superior, inferior, and posterior margins of the lesion. The adhesion between the gastric fundus and the left side of the lesion was relatively dense. After dissection, the lesion was completely resected using a Hem-o-Lock clip for vessel occlusion, and an intact capsule was observed. After removal of the retroperitoneal adipose tissue, one left renal hilar lymph node (1.5 cm × 1 cm × 0.8 cm) was excised. The operation went smoothly. The patient’s vital signs remained stable throughout the procedure, and the patient recovered uneventfully postoperatively and was discharged on March 1, 2024.

### 2.5 Pathological findings

Gross Specimen: The retroperitoneal tumor consisted of a piece of gray-white and gray-red tissue, measuring approximately 6 cm × 5 cm × 3 cm. The cut surface was gray-white with a firm consistency, and focal calcifications were observed. One left renal hilar lymph node was submitted, measuring approximately 1.5 cm × 1 cm × 0.8 cm.

Microscopic Examination: Lymphoproliferative lesion with preserved follicular architecture. Some follicles exhibited regressed/atretic germinal centers. The interfollicular zones showed dense infiltration by plasma cells (**Figures 3B–L**).

Immunohistochemistry: B-cell markers: CD20 (+), CD79α (+); T-cell markers: CD3 (+), CD5 (+); Germinal center B-cell markers: CD10 (+), BCL6 (+), and BCL2 (-) in germinal centers, Cyclin D1(-); FDC marker: CD21 showed preserved FDC networks; Proliferation index: Ki-67 approximately 90% in germinal centers; Plasma cell markers: CD43 (+), CD38 (+), CD138 (+), MUM1 (+) in interfollicular regions; HHV-8 (-); Kappa/lambda light chains: no light chain restriction, confirming polyclonal plasma cell proliferation.

Molecular Pathology: B-cell clonality analysis revealed no evidence of monoclonal immunoglobulin gene rearrangement.

Pathological Diagnosis: Retroperitoneal tumor: Consistent with plasma cell type Castleman disease, with focal calcifications. Left renal hilar lymph node: Reactive hyperplasia.

### 2.6 Follow-up

**Figure 4:**
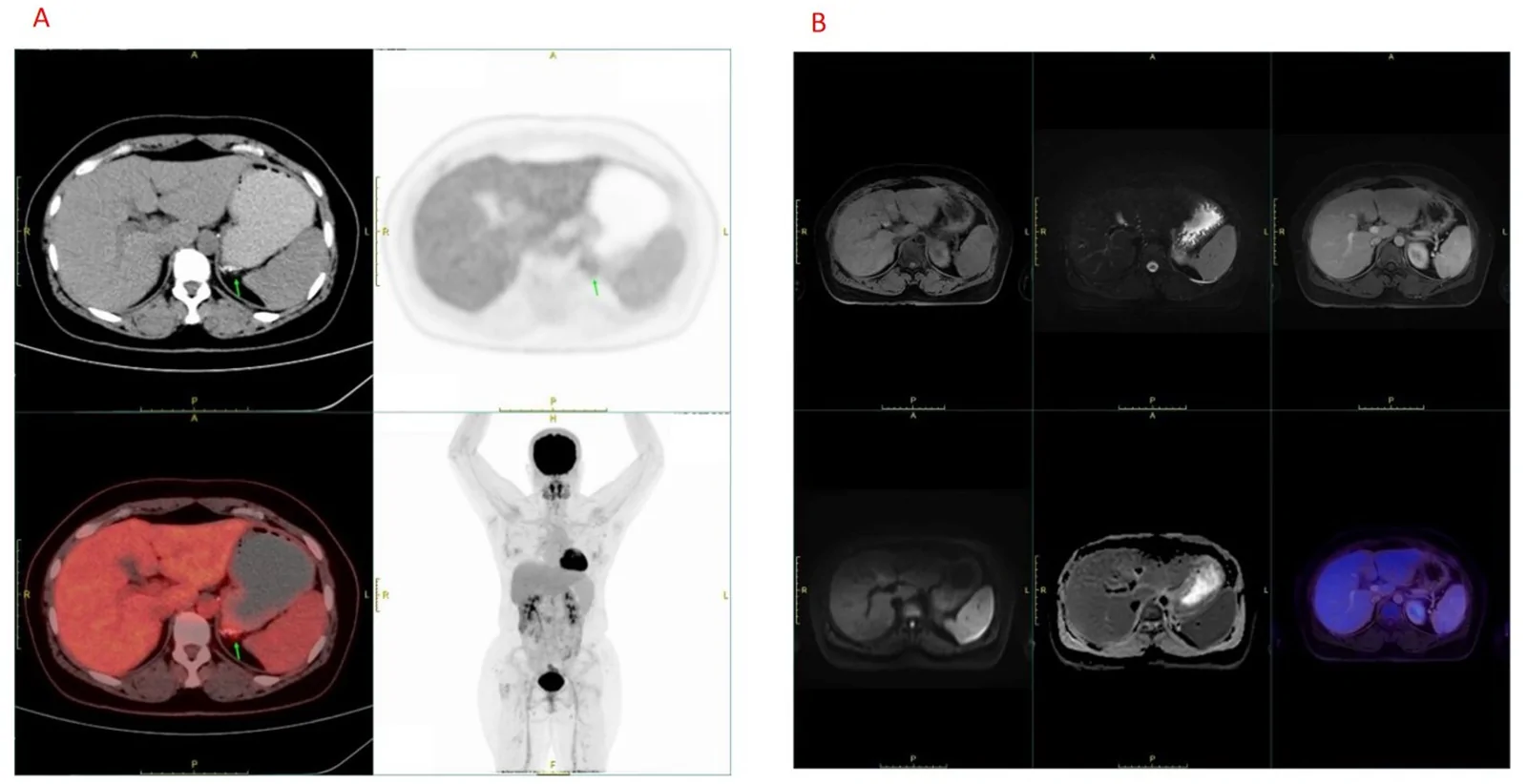
Postoperative follow-up functional imaging examinations at the early postoperative stage. **(A)** Follow-up PET/CT: Only postoperative benign changes were observed in the left adrenal region, without detectable abnormally increased radiotracer uptake suggestive of residual or recurrent lesions. **(B)** Follow-up PET/MRI: The left adrenal gland was surgically resected. No abnormal imaging signals or pathological radiotracer accumulation were identified within the surgical bed.

The patient recovered smoothly after surgery, with only mild incisional discomfort, which resolved gradually with symptomatic treatment. On May 21, 2024, follow-up chest and abdominal CT showed postoperative changes in the left adrenal region, with no evidence of tumor recurrence or complications requiring intervention. On May 24, 2024, whole-body ¹^8^F-FDG PET/CT (**Figure 4A**) and PET/MRI (**Figure 4B**) demonstrated postoperative changes in the left adrenal region, with no abnormal ¹^8^F-FDG uptake in the surgical area; no enlarged lymph nodes or hypermetabolic lesions were observed throughout the body. At the 2-year follow-up, the patient’s hyperhidrosis and facial flushing had markedly improved compared with the preoperative status, and no other specific complaints were reported.

## 3 Discussion

### 3.1 Rarity and diagnostic challenges

CD is an uncommon a heterogeneous group of lymphoproliferative disorders with substantial clinicopathological heterogeneity. Population-based and claims-based studies have shown that CD remains a rare disease overall, with an estimated prevalence of approximately 21–25 cases per million (13). Among these entities, UCD is the most frequent clinical form, whereas HHV-8-associated multicentric CD and idiopathic multicentric CD (iMCD) are less common but usually more aggressive systemic disorders (14). Multiple studies have demonstrated that the hyaline vascular subtype accounts for nearly 90% of UCD cases (15, 16), and Wang Z et al. have reported a case of hyaline vascular UCD arising in the left adrenal region (12). UCD can occur across a wide age range and has been reported most often in the mediastinum, cervical region, abdomen, and retroperitoneum (11), and the adrenal region accounts for approximately 12% of retroperitoneal UCD (9). Consistent with previous literature, adrenal-region UCD is easily misdiagnosed as other adrenal and periadrenal tumors. Because the adrenal region is more commonly occupied by adrenocortical adenoma, pheochromocytoma, ganglioneuroma, metastatic disease, lymphoma, or retroperitoneal soft-tissue tumors, CD is generally not prioritized in the initial differential diagnosis. This diagnostic challenge is compounded when endocrine evaluation is unremarkable, as in many reported adrenal UCD cases, because the lesion then appears to be a nonfunctional adrenal mass rather than a lymphoid proliferation. Therefore, our findings further support the view that UCD should be included in the differential diagnosis of well-circumscribed adrenal-region masses, especially when biochemical testing does not support a hormonally active adrenal neoplasm.

### 3.2 Imaging insights

Radiologic assessment plays a central role in the preoperative evaluation of adrenal-region masses, but imaging findings of UCD are not sufficiently specific to establish a definitive diagnosis. Previous reports have described UCD lesions on computed tomography (CT) as well-defined, hypervascular soft-tissue masses with avid or heterogeneous enhancement, sometimes accompanied by feeding vessels or calcification (9, 17). Magnetic resonance imaging (MRI) findings are likewise variable, although many lesions show intermediate T1 signal intensity and heterogeneous or hyperintense T2 signal with strong post-contrast enhancement (18, 19). Functional imaging may demonstrate fluorodeoxyglucose uptake on positron emission tomography (PET), but this feature does not distinguish CD from malignant lesions (20). The imaging characteristics in the present case are broadly in line with these prior observations, in that the lesion presented as a localized adrenal-region mass requiring exclusion of more common neoplastic entities. This is consistent with the broader literature emphasizing that radiologic findings in UCD are suggestive rather than diagnostic. Compared with malignant adrenal lesions, UCD often demonstrates indolent behavior and a clear interface with surrounding tissues, but overlap remains considerable. In contrast to pheochromocytoma or paraganglioma, patients with adrenal-region UCD may lack hypertension, catecholamine excess, or characteristic biochemical abnormalities. Similarly, unlike lymphoma, UCD is usually solitary and does not necessarily involve widespread nodal disease or constitutional symptoms (21). The presence of linear and punctate calcifications with prominent contrast enhancement, as seen in our case, places Castleman disease in the differential diagnosis of calcified retroperitoneal masses, alongside teratomas, neurogenic tumors, and chronic hematomas. The present case therefore reinforces the need for a multidisciplinary diagnostic approach integrating imaging, endocrinologic testing, surgical assessment, and final histopathology.

### 3.3 Histopathology

Histologically, CD has traditionally been divided into hyaline vascular, plasma cell, and mixed variants, although modern classification increasingly emphasizes clinicopathologic context over rigid morphologic subtyping alone (11, 22). In the present case, the diagnosis of plasma cell type UCD was established on the basis of localized disease and pathological features consistent with CD. Plasma cell type lesions typically show hyperplastic or regressed germinal centers, preserved to expanded mantle zones, interfollicular plasmacytosis, and varying degrees of vascular proliferation (23). This pattern differs from the classic hyaline vascular appearance dominated by atretic germinal centers, onion-skin mantle zones, and penetrating hyalinized vessels. These findings support earlier observations that plasma cell-rich histology may occur in UCD and does not necessarily imply multicentric disease or systemic inflammatory activity (24). A major issue in such cases is the distinction from pathological mimics. Reactive lymphadenopathy secondary to chronic infection may also show plasmacytosis, while IgG4-related disease can present with plasma cell infiltration in retroperitoneal sites. Likewise, marginal zone lymphoma, plasmacytoma, and other lymphoid neoplasms may enter the differential diagnosis if architecture is distorted or clonality is suspected. In the present case, lymphoma was excluded based on preserved follicular architecture, polyclonal plasma cell proliferation confirmed by κ and λ light chain staining without light chain restriction, and the absence of BCL2 overexpression in germinal centers.

### 3.4 Systemic manifestations and the role of IL-6

The present case exhibited a pronounced systemic inflammatory profile—elevated IL-6 (11.21 pg/mL), ESR, and CA724, splenomegaly (123.5 mm), and long-standing intermittent hyperhidrosis and facial flushing—that is more characteristic of multicentric disease. Following complete resection, IL-6 normalized to 2.63 pg/mL and ESR fell to 3 mm/h, providing clinical evidence for the IL-6-driven hypothesis of systemic manifestations in UCD. However, spleen size remained largely unchanged (125.3 mm), suggesting that splenomegaly in this case may not have been driven solely by IL-6, or that a longer interval may be required for regression. This observation underscores the complexity of the inflammatory milieu in CD and cautions against oversimplifying the causal relationship between IL-6 and organomegaly.

The differential diagnosis between UCD with systemic features and iMCD is of critical importance. This case does not meet the diagnostic criteria for iMCD: the patient had no TAFRO-like features (thrombocytopenia, anasarca, fever, renal dysfunction, or organomegaly beyond regional lymphadenopathy); the lymphadenopathy was regionally confined to the retroperitoneum; postoperative IL-6 normalized and remained normal at two-year follow-up with negative PET-CT and PET-MRI; and HHV-8 was negative both serologically and immunohistochemically. Furthermore, autoimmune conditions were excluded: ANA was <1:80 (negative), RF was 12 IU/L (within normal limits), and syphilis and HIV serology were negative. The patient had no clinical features suggestive of systemic lupus erythematosus. These findings support the diagnosis of UCD over iMCD.

### 3.5 Treatment and prognosis

For UCD, complete surgical excision remains the standard treatment and is strongly recommended whenever anatomically feasible (7). The rationale is twofold: surgery provides definitive histological diagnosis and is usually curative. Large institutional and systematic analyses have shown excellent outcomes after complete resection, with very high disease control rates and minimal long-term morbidity in localized disease (25). The favorable postoperative course in the present patient is therefore highly consistent with prior evidence and further confirms the effectiveness of surgery in adrenal-region UCD. This case also highlights an important practical issue: surgery is often undertaken not only for treatment but also because preoperative exclusion of malignancy is difficult. In cases where complete resection is not feasible, other options have been described, including partial resection, radiotherapy, or medical therapy tailored to symptoms and residual disease burden (25). These approaches are more relevant to unresectable UCD or multicentric forms than to the present case. Nonetheless, their existence underscores the importance of accurately distinguishing UCD from MCD, as the latter requires fundamentally different management, often involving anti–IL-6 strategies, rituximab-based approaches, or systemic therapy (26–28).

### 3.6 Limitations and future directions

Several limitations of this study should be acknowledged. First, this is a single case report, and the rarity of the disease inherently limits generalizability. Although case reports are valuable for documenting unusual presentations, they cannot define incidence, risk factors, or optimal surveillance strategies. Second, the duration of follow-up, while reassuring, remains relatively limited for assessing very late recurrence. Longer-term observation would provide stronger prognostic evidence. Third, as with many retrospective case descriptions, the preoperative diagnostic pathway was constrained by the nonspecific nature of available imaging and laboratory findings. Furthermore, not all ancillary biomarkers of research interest in CD were necessarily informative in localized disease. Fourth, preoperative whole-body PET-CT was not performed, which limited the initial systemic assessment; however, postoperative PET-CT and PET-MRI at three months confirmed the absence of disseminated disease.

Future studies should focus on systematic aggregation of adrenal and retroperitoneal UCD cases through multicenter registries or pooled analyses. Such efforts could clarify whether adrenal-region UCD has distinctive clinical, radiologic, or histopathologic characteristics compared with UCD at other anatomical sites. Standardized reporting of imaging features, immunophenotypic findings, operative details, and long-term outcomes would also improve diagnostic recognition and management. In addition, further research is warranted to explore whether plasma cell type UCD in unusual locations carries any differential risk of recurrence, occult systemic inflammation, or progression compared with the more commonly reported hyaline vascular subtype.

## 4 Conclusion

This case report describes plasma cell type UCD arising in the adrenal region, a condition that poses diagnostic challenges due to its nonspecific endocrine and imaging findings. Based on available literature, Plasma cell type UCD arising in the adrenal region appears to be uncommon. The clinicopathological findings from the present case enrich the existing literature and offer further insights into the diagnosis and management of this disorder. The patient presented with long-standing intermittent hyperhidrosis and facial flushing. Imaging revealed a hypervascular mass in the left adrenal region, while endocrine workup showed no evidence of adrenal hormone hypersecretion. The mass was completely resected via laparoscopic surgery, and postoperative pathology with immunohistochemistry confirmed the diagnosis of plasma cell type UCD. The patient’s symptoms improved markedly after surgery, and no recurrence or metastasis was observed during two years of follow-up. This case suggests that UCD should be considered in the differential diagnosis of well-circumscribed adrenal region masses without endocrine abnormalities. The clinicopathological features documented in this case may contribute to the body of literature and provide deeper insights into the diagnosis and management of this condition. Complete surgical resection achieved a favorable outcome in this patient. However, as this study is a single case report, the generalizability of its conclusions is limited. Future multicenter case series is needed to further clarify the clinical characteristics and optimal management strategies for UCD in this location.

## Data Availability

The original contributions presented in the study are included in the article/supplementary material. Further inquiries can be directed to the corresponding authors.
